# All-Trans Retinoic Acid Attenuates Transmissible Gastroenteritis Virus-Induced Apoptosis in IPEC-J2 Cells via Inhibiting ROS-Mediated P_38_MAPK Signaling Pathway

**DOI:** 10.3390/antiox11020345

**Published:** 2022-02-10

**Authors:** Junning Pu, Daiwen Chen, Gang Tian, Jun He, Zhiqing Huang, Ping Zheng, Xiangbing Mao, Jie Yu, Junqiu Luo, Yuheng Luo, Hui Yan, Bing Yu

**Affiliations:** Key Laboratory for Animal Disease-Resistance Nutrition, Ministry of Education/Institute of Animal Nutrition, Sichuan Agricultural University, Chengdu 611130, China; junningpu@163.com (J.P.); chendwz@sicau.edu.cn (D.C.); tgang2008@126.com (G.T.); hejun8067@163.com (J.H.); zqhuang@sicau.edu.cn (Z.H.); zpind05@163.com (P.Z.); acatmxb2003@163.com (X.M.); yujie@sicau.edu.cn (J.Y.); junqluo2018@tom.com (J.L.); yhluo@sicau.edu.cn (Y.L.); yan.hui@sicau.edu.cn (H.Y.)

**Keywords:** all-trans retinoic acid, transmissible gastroenteritis virus, apoptosis, oxidative stress, ROS/P_38_MAPK pathway, IPEC-J2 cells

## Abstract

Transmissible gastroenteritis virus (TGEV) can cause diarrhea, dehydration, and high mortality in piglets, which is closely related to intestinal epithelial cell apoptosis caused by TGEV infection. All-trans retinoic acid (ATRA) is the active metabolite of vitamin A, which has antioxidant and anti-apoptotic properties. However, it is unknown whether ATRA can attenuate TGEV-induced IPEC-J2 cells apoptosis. Therefore, we investigated the protective effects of ATRA on TGEV-induced apoptosis of IPEC-J2 cells and explored the potential molecular mechanism. Our results indicated that TGEV infection caused IPEC-J2 cells damage and apoptosis. However, ATRA treatment attenuated TGEV-induced IPEC-J2 cells damage by upregulating the mRNA expressions of ZO-1, Occludin, and Mucin-1. ATRA treatment also attenuated TGEV-induced apoptosis in IPEC-J2 cells by downregulating the expression of Caspase-3, which is related to the inhibition of death receptor (Fas and Caspase-8) and mitochondrial (Bax, Bcl-2, and Caspase-9) pathways. Moreover, ATRA treatment prevented TGEV-induced ROS and MDA production and the upregulation of P_38_MAPK phosphorylation level, which is related to the increase in the activities of antioxidant enzymes (SOD, CAT, and T-AOC) and the mRNA abundance of antioxidant-related genes (GPX1, GPX2, SOD1, CAT, GCLC, and GCLM). In addition, treatment of TGEV-infected IPEC-J2 cells with the ROS inhibitors (NAC) significantly reduced the protein levels of p-P_38_MAPK, Fas, Bax, and Cleaved-caspase-3 and the percentage of apoptotic cells. Our results indicated that ATRA attenuated TGEV-induced apoptosis in IPEC-J2 cells via improving the antioxidant capacity, thereby inhibiting the cell damage. the mechanism of which is associated with the inhibition of ROS-mediated P_38_MAPK signaling pathway.

## 1. Introduction

Transmissible gastroenteritis virus (TGEV) is a coronavirus with a single-stranded positive-sense RNA genome [1]. TGEV replicates in enterocytes covering porcine small intestinal villi, provokes villous atrophy and cryptal cells hyperplasia, causing diarrhea, dehydration and vomiting in piglets and resulting in significant economic losses to the global pig industry [2]. Consistent with pathological changes in vivo, TGEV infection also induces the cytopathic effects (CPE) of host cells in vitro [3,4], which may be related to apoptosis induced by TGEV infection. Apoptosis is a strictly regulated cell-death mechanism that plays a crucial role not only in the normal development of cells but also in the pathogenesis of viral infections [5,6]. A large number of studies have reported infections with swine enteropathogenic coronaviruses such as porcine epidemic diarrhea virus (PEDV) [7], porcine delta coronavirus (PDCoV) [8], and swine acute diarrhea syndrome coronavirus (SADS-CoV) [9], resulted in host cell apoptosis. Previous studies have also shown that TGEV infection can activate pro-apoptotic signals, trigger apoptosis, and further lead to CPE and cell death of host cells [10,11,12]. The evidence of TGEV-induced apoptosis indicates that apoptosis is involved in the pathogenesis of TGEV.

Oxidative stress is a state in which the imbalance between the antioxidant system and oxidative system leads to excessive production of reactive oxygen species (ROS) [13]. ROS is a known toxic product of cellular metabolism and is participated in the regulation of various pathological processes such as autophagy, inflammation, and apoptosis [14]. Many studies have shown that ROS participates in virus-induced apoptosis. For example, Enterovirus 71 infection caused Vero cells apoptosis by mediating ROS accumulation [15]. Porcine parvovirus infection caused ST cells apoptosis by activating ROS-mediated mitochondrial apoptosis pathway [16]. Ding et al. reported that TGEV infection induced PK-15 cells apoptosis through ROS-mediated AIF pathway [17]. Furthermore, ROS is also related to some apoptosis pathways, such as P_38_MAPK and p53 pathways [18]. Xu et al. reported that PEDV infection induced Vero cells apoptosis via ROS-mediated p53 signaling pathway [19]. Previous studies have shown that TGEV infection can cause the accumulation of ROS in PK-15 cells, which could activate the P_38_MAPK and p53 pathways and induce apoptosis [20].

It is well known that vitamin A is an essential fat-soluble micronutrient for maintaining human and all mammalian health [21]. All-trans retinoic acid (ATRA) is the major active metabolite of vitamin A and regulates a range of biological processes including anti-cancer, anti-inflammatory, vision, and cell proliferation and differentiation [22,23]. Furthermore, previous studies reported that ATRA has antioxidant and anti-apoptotic properties. Rao et al. reported that ATRA alleviated hepatic ischemia/reperfusion injury by enhancing the activity of superoxide dismutase (SOD) and inhibiting malondialdehyde (MDA) formation in rats [24]. Khafaga et al. reported that ATRA ameliorated doxorubicin-induced apoptosis through suppressing the mitochondrial apoptotic pathway [25]. Choudhary et al. reported that ATRA prevented angiotensin II and mechanical stretch-induced cardiomyocyte apoptosis by inhibiting ROS production and increasing the antioxidant defense system [26]. In addition, previous studies reported that ATRA can attenuate arsenic-induced oxidative stress and apoptosis via regulating MAPK pathway [27]. These results shown that ATRA may play a key role in antioxidant and anti-apoptosis. However, whether ATRA can attenuate TGEV-induced apoptosis remains unclear.

Here, based on the model of TGEV-infected intestinal porcine epithelial cells (IPEC-J2) [28], we aimed to study the protective potential of ATRA on TGEV-induced apoptosis in IPEC-J2 cells and to explore the underlying molecular mechanism. We hypothesized that ATRA might attenuate TGEV-induced IPEC-J2 cells apoptosis via inhibiting ROS-mediated P_38_MAPK pathway.

## 2. Materials and Methods

### 2.1. Reagents and Antibodies

All-trans retinoic acid (ATRA, ≥98% HPLC), dimethyl sulfoxide (DMSO), and 2′,7′-dichlorofluorescein-diacetate (DCFH-DA) were acquired from Sigma-Aldrich (Shanghai, China). N-Acetyl-L-cysteine (NAC) was acquired from Beyotime Biotechnology (Shanghai, China). Primary antibodies against Fas, Caspase-3, Caspase-8, and Caspase-9 were purchased from Cell Signaling Technology (Beverly, MA, USA). P_38_MAPK and phospho-P_38_MAPK were obtained from ZEN Bioscience (Chengdu, China). β-actin, B-cell lymphoma 2 (Bcl-2), Bcl-2-associated X (Bax), and secondary antibodies were obtained from Santa Cruz (Santa Cruz, CA, USA).

### 2.2. Cells Culture and Virus Infection

IPEC-J2 cells obtained from the American Type Culture Collection (ATCC, Manassas, VA, USA) and cultured in Dulbecco’s modified Eagle’s medium and Ham’s F-12 nutrient mixture (DMEM/F12, Gibco, Shanghai, China) supplemented with 10% fetal bovine serum (Gibco, Shanghai, China) and 1% streptomycin and penicillin (Gibco, Shanghai, China) at 37 °C with a humidified 5% CO2 incubator. TGEV (SC-T strain) was provided by Prof. Zhiwen Xu, Sichuan Agricultural University. TGEV was propagated in swine testicle (ST) cells and virus titers were measured by TCID50 [29]. For TGEV infection, confluent (70–80%) IPEC-J2 cells were infected with TGEV (MOI = 1) for 1 h at 37 °C. After that, cells were washed 3 times with PBS and cultured in fresh growth medium.

### 2.3. Cell Viability Assay

IPEC-J2 cells were cultured in 96-well plates (1 × 10^4^ cells per well) and treated with various concentrations of ATRA (0, 1, 10, 20, 40, 60, 80, 100, and 200 μM) at 37 °C for 36 h to determine the non-toxic concentration of ATRA. Furthermore, IPEC-J2 cells were infected with TGEV (1 MOI) for 1 h, followed by incubation with various concentrations of ATRA (0, 1, 10, 20, 40, 60, and 80 μM) for 36 h to study the protective effects of ATRA against TGEV. Finally, cell viability was evaluated using the CCK-8 assay (Beyotime Biotechnology, Shanghai, China). Briefly, cells were incubated with 10 ul CCK-8 assay reagent for 2 h at 37 °C, and the optical density (OD) was measured at 450 nm with a microplate reader (SpectraMax M2, Sunnyvale, CA, USA).

### 2.4. Determination of LDH Activity and Antioxidant Capacity

Lactate dehydrogenase (LDH) activity in cell culture medium was determined by using LDH assay kit (Nanjing Jiancheng Bioengineering Institute, Nanjing, China) according to the manufacturer’s instructions. The activities of glutathione peroxidase (GPx), superoxide dismutase (SOD), catalase (CAT), total antioxidant capacity (T-AOC), and the level of malondialdehyde (MDA) in IPEC-J2 cells were determined by using the commercial kits (Nanjing Jiancheng Bioengineering Institute, Nanjing, China) combined with a UV–VIS spectrophotometer (UV1100, MAPADA, Shanghai, China) according to the manufacturer’s instructions. The total protein concentration in IPEC-J2 cells was measured by a BCA protein assay kit (Thermo Scientific, Waltham, MA, USA).

### 2.5. Determination of Intracellular ROS

The intracellular ROS level was determined by using DCFH-DA (Sigma-Aldrich; D6883). Briefly, IPEC-J2 cells were mock-infected or infected with TGEV (1 MOI) for 1 h, followed by incubation with or without 80 µM ATRA at 37 °C for 36 h. Then, cells were digested with 0.25% trypsin without EDTA, washed with PBS and incubated with 100 μL DCFH-DA (10 µM) at 37 °C for 30 min in the dark. After incubation, cells were washed thrice with serum-free cell culture medium and resuspended in 400 μL of PBS, and the DCF fluorescence intensity of 10,000 cells was measured by a BD FACSVerseTM flow cytometer (BD Biosciences, East Rutherfor, NJ, USA). The DCF fluorescence intensity indicated the concentration of intracellular ROS, and the result was analyzed using FlowJo software (Version 10.5.3, FlowJo LLC, Ashland, OR, USA).

### 2.6. Flow Cytometric Determination of Apoptosis

IPEC-J2 cell apoptosis was measured using the Annexin V-FITC apoptosis detection kit (BioLegend, San Diego, CA, USA). Briefly, cells were collected, resuspended in 100 μL 1 × binding buffer, and stained with 3 μL Annexin V-FITC for 20 min at room temperature in the dark. Then, cells were resuspended in 400 μL 1 × binding buffer, and 1 μL propidium iodide (PI, 1 mg/mL) per test was added. Finally, 10,000 cells were acquired, and apoptosis was measured using a BD FACSVerseTM flow cytometer (BD Biosciences, USA). All data were analyzed using FlowJo software (Version 10.5.3, FlowJo LLC).

### 2.7. Real-Time Quantitative PCR

Total RNA was extracted using TRIzol reagent (Invitrogen, Shanghai, China). Then, cDNA was synthesized from 1 µg total RNA using the PrimeScripte RT kit (TaKaRa Biotechnology Co., Ltd., Dalian, China) according to the manufacturer’s instructions. Real-time quantitative PCR was performed using SYBR Premix Ex Taq^TM^ kits (TaKaRa) on the CFX96 Real-Time PCR Detection System (Bio-Rad, Richmond, CA, USA). All primers used in this study were listed in Supplemental Table S1. The 2^−ΔΔCt^ method was used to analyze the relative mRNA levels of target genes, and β-actin was used as the house-keeping gene [30].

### 2.8. Western Blot Analysis

Proteins were extracted from IPEC-J2 cells with RIPA lysis buffer (containing PMSF and phosphatase inhibitor) and the protein concentrations in the supernatants were measured using a BCA protein assay reagent (Thermo Scientific, MA, USA). Samples containing equal amounts of protein (25 μg) were subjected to sodium dodecyl sulphate–polyacrylamide gel electrophoresis (SDS-PAGE) and subsequently transferred to polyvinylidene difluoride (PVDF) membranes (Merck Millipore Ltd., Tullagreen, Ireland). The membranes were blocked with 5% nonfat dry milk for 1 h at room temperature, and then incubated overnight at 4 °C with indicated primary antibodies, followed by incubating with the corresponding HRP-conjugated secondary antibodies for 1 h at room temperature. The blots were visualized in the ChemiDoc^TM^ XRS Imager System (Bio-Rad) and quantified by using Image Lab software (Bio-Rad). The ratio of the densitometric values of the target protein to the reference protein is calculated and expressed as a relative level to control value.

### 2.9. Statistical Analysis

All data were expressed as the means ± standard error of mean (SEM). The normality of data was determined by the Shapiro–Wilk test, and the statistical differences were analyzed by the unpaired two-tailed Student’s *t*-test and/or one-way analysis of variance (ANOVA) using SPSS 22.0 statistics software (Chicago, IL, USA). Differences were considered significant at *p* < 0.05.

## 3. Results

### 3.1. Effects of ATRA and TGEV on the Viability of IPEC-J2 Cells

**Figure 1:**
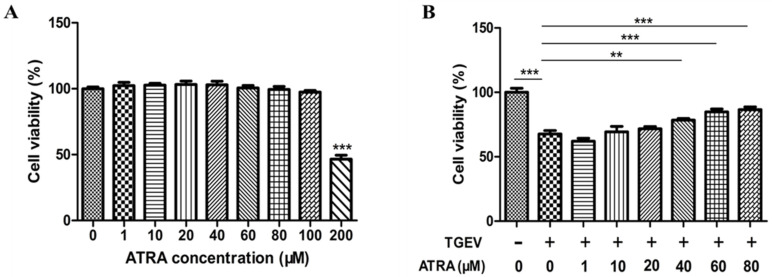
Effects of ATRA and TGEV on the viability of IPEC-J2 cells were measured by CCK8 reagent. (**A**) IPEC-J2 cells were exposed to ATRA at different concentrations (0, 1, 10, 20, 40, 60, 80, 100, and 200 μM) for 36 h to determine the non-toxic concentration of ATRA (*n* = 8). *** *p* < 0.001 compared with the control group (0 μM ATRA). (**B**) IPEC-J2 cells were infected with TGEV (1 MOI) for 1 h, followed by incubation with various concentrations of ATRA (0, 1, 10, 20, 40, 60, and 80 μM) for 36 h to study the protective effects of ATRA against TGEV (*n* = 8). ** *p* < 0.01 and *** *p* < 0.001.

To measure the safe dose of ATRA, IPEC-J2 cells were exposed to ATRA at different concentrations (0, 1, 10, 20, 40, 60, 80, 100, and 200 μM) for 36 h. The viability of IPEC-J2 cells significantly decreased at ATRA concentrations above 100 μM compared with the control group (*p* < 0.001) (Figure 1A). Therefore, the ATRA concentration range used in subsequent experiments was 0~80 μM. Next, we investigated the protective effects of ATRA on TGEV-infected IPEC-J2 cells. As shown in Figure 1B, after TGEV infects IPEC-J2 cells, the cell viability was significantly reduced (*p* < 0.001). However, when TGEV-infected IPEC-J2 cells were treated with 40~80 μM of ATRA for 36 h, there was a dose-dependent increase in cell viability (*p* < 0.01).

### 3.2. ATRA Attenuated TGEV-Induced IPEC-J2 Cells Damage

**Figure 2:**
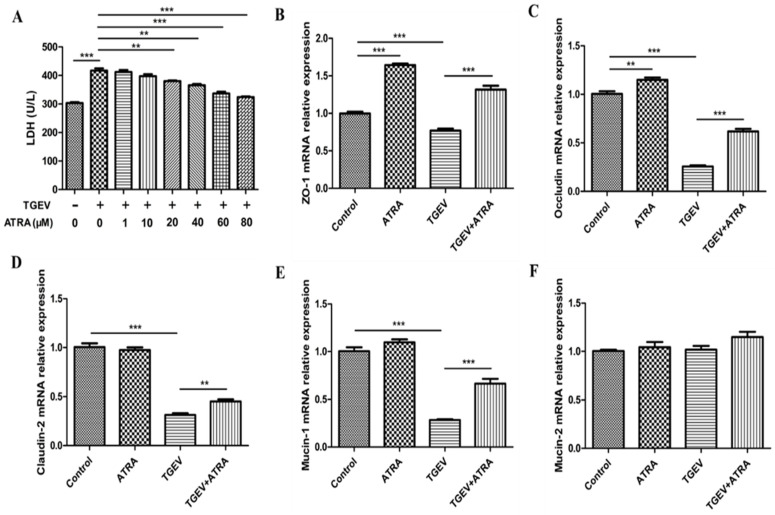
The protective effects of ATRA on the damage of TGEV-induced IPEC-J2 cells. (**A**) IPEC-J2 cells were infected with TGEV (1 MOI) for 1 h, followed by incubation with various concentrations of ATRA (0, 1, 10, 20, 40, 60, and 80 μM) for 36 h. LDH viability was measured by the LDH assay (*n* = 4). (**B**–**F**) The cells were infected with or without TGEV (1 MOI) for 1 h, followed by incubation with or without 80 μM ATRA for 36 h. The mRNA abundance of ZO-1, Occludin, Claudin-2, Mucin-1, and Mucin-2 in IPEC-J2 cells were analyzed by real-time PCR (*n* = 4). ** *p* < 0.01 and *** *p* < 0.001.

In order to determine whether ATRA attenuated the IPEC-J2 cells damage induced by TGEV, LDH activity in the IPEC-J2 cell culture medium was tested. LDH release is an important parameter to assess the severity of cells damage. As shown in Figure 2A, there was a marked rise of the activity of LDH in the TGEV group compared with the control group. However, compared with the TGEV group, ATRA treatment significantly reduced the LDH activity in a dose-dependent manner with the most effective at a concentration of 80 μM ATRA (*p* < 0.01). Therefore, the working concentration of ATRA in the following experiments was set to 80 μM. To further determine the effects of ATRA on TGEV--induced cells damage, we detected the mRNA abundance of tight junctions-related genes (Figure 2B–F). Compared with the control group, the mRNA abundances of ZO-1 and Occludin were significantly increased in the ATRA group (*p* < 0.01). TGEV infection significantly downregulated the mRNA expressions of ZO-1, Occludin, Claudin-2, and Mucin-1 (*p* < 0.001). ATRA treatment significantly reversed this downregulation of ZO-1, Occludin, Claudin-2, and Mucin-1 mRNA expressions induced by TGEV (*p* < 0.01). However, Mucin-2 mRNA expression did not differ among groups *(p* > 0.05).

### 3.3. ATRA Attenuated TGEV-Induced Apoptosis in IPEC-J2 Cells

**Figure 3:**
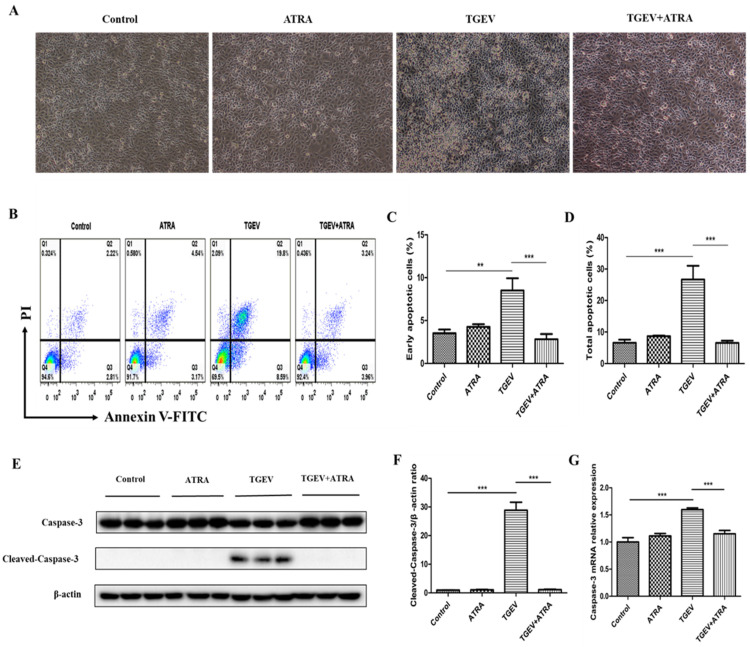
Effects of ATRA on the apoptosis in TGEV-infected IPEC-J2 cells. The cells were infected with or without TGEV (1 MOI) for 1 h, followed by incubation with or without 80 μM ATRA for 36 h. (**A**) The morphological changes of IPEC-J2 cells were observed by inverted microscope (Scale bars, 50 μm). (**B**) Annexin V-FITC/PI staining cells were analyzed by flow cytometry. (**C**) Quantification of the percentages of early apoptotic cells (Q3 quadrant, *n* = 4). (**D**) Quantification of the percentages of total apoptotic cells (Q3+Q2 quadrants, *n* = 4). (**E**,**F**) Cleaved-caspase-3 protein level was analyzed by Western blot (*n* = 3). (**G**) Caspase-3 mRNA expression was analyzed by real-time PCR (*n* = 4). ** *p* < 0.01 and *** *p* < 0.001.

Apoptosis occurs in the TGEV infection-induced cytopathic effect (CPE) and cell death [31]. To assess whether ATRA attenuated TGEV-induced apoptosis in IPEC-J2 cells, we firstly observed the morphological changes of IPEC-J2 cells. As shown in Figure 3A, compared with the control group, the cytopathic effect appeared in TGEV-infected IPEC-J2 cells. ATRA treatment could evidently reverse the CPE induced by TGEV. To further confirm the effects of ATRA on TGEV-induced apoptosis, apoptotic cells were detected by flow cytometry after Annexin V-FITC and PI staining. As shown in Figure 3B–D, the number of early and total apoptotic IPEC-J2 cells was markedly increased by TGEV infection (*p* < 0.01). However, ATRA treatment significantly suppressed IPEC-J2 cell apoptosis induced by TGEV (*p* < 0.001). Moreover, TGEV infection significantly increased the Cleaved-caspase-3 protein level and Caspase-3 mRNA expression compared with the control group (*p* < 0.001). However, ATRA treatment significantly reversed this elevation of Cleaved-caspase-3 protein level and Caspase-3 mRNA expression induced by TGEV (*p* < 0.001) (Figure 3E–G).

### 3.4. ATRA Attenuated TGEV-Induced Apoptosis in IPEC-J2 Cells through Regulating Death Receptor and Mitochondrial Pathways

**Figure 4:**
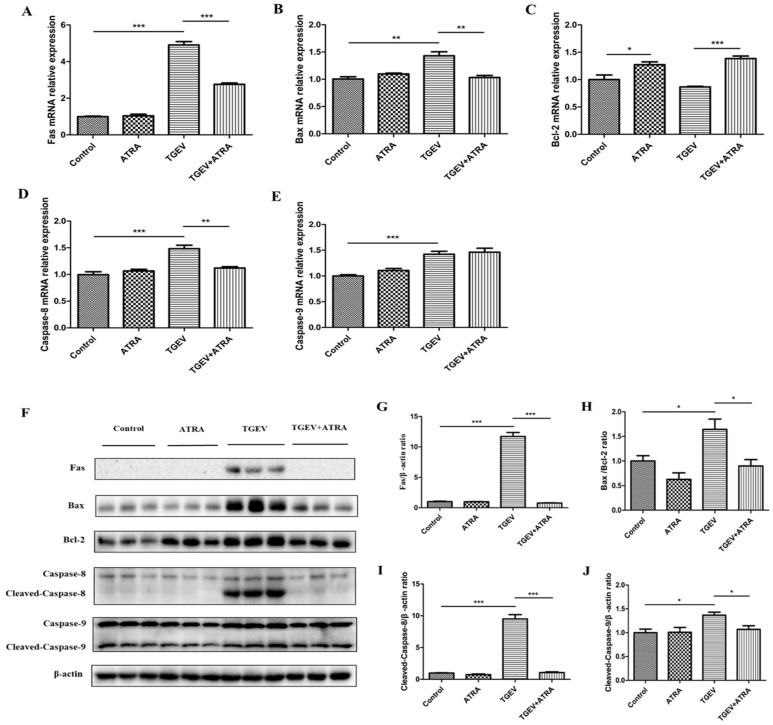
Effects of ATRA on death receptor and mitochondria pathways in TGEV-infected IPEC-J2 cells. The cells were infected with or without TGEV (1 MOI) for 1 h, followed by incubation with or without 80 μM ATRA for 36 h. (**A**–**E**) The mRNA abundance of Fas, Bax, Bcl-2, Caspase-8, and Caspase-9 in IPEC-J2 cells were analyzed by real-time PCR (*n* = 4). (**F**–**J**) The levels of apoptotic-related proteins in IPEC-J2 cells were analyzed by Western blot (*n* = 3). * *p* < 0.05, ** *p* < 0.01 and *** *p* < 0.001.

To gain insight into the mechanism of ATRA-attenuated TGEV-induced apoptosis in IPEC-J2 cells, the expressions of apoptotic-related genes involved in the death receptor (Fas and Caspase-8) and mitochondrial (Bax, Bcl-2 and Caspase-9) pathways were analyzed by real-time PCR. As shown in Figure 4A–E, TGEV infection significantly increased the mRNA expressions of Fas, Bax, Caspase-8, and Caspase-9 in IPEC-J2 cells (*p* < 0.05). However, ATRA treatment significantly decreased the mRNA expressions of Fas, Bax, and Caspase-8, and increased Bcl-2 mRNA expression compared with the TGEV group (*p* < 0.05). To further confirm the effects of ATRA on the apoptotic pathways in TGEV-infected IPEC-J2 cells, we detected the levels of apoptotic-related proteins in IPEC-J2 cells. As shown in Figure 4F–J, TGEV infection significantly upregulated the protein levels of Fas, Cleaved-caspase-8, Cleaved-caspase-9, and the ratio of Bax/Bcl-2 in IPEC-J2 cells (*p* < 0.05), while ATRA treatment significantly prevented the upregulation of Fas, Cleaved-caspase-8 and Cleaved-caspase-9 protein levels, and the ratio of Bax/Bcl-2 induced by TGEV (*p* < 0.05).

### 3.5. ATRA Attenuated TGEV-Induced ROS Production in IPEC-J2 Cells

**Figure 5:**
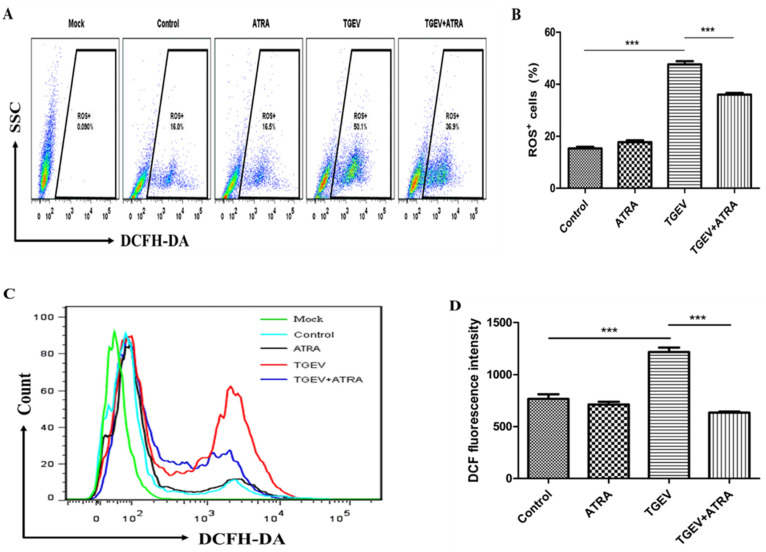
Effects of ATRA on the level of ROS in TGEV-infected IPEC-J2 cells. The cells were infected with or without TGEV (1 MOI) for 1 h, followed by incubation with or without 80 μM ATRA for 36 h. (**A**) ROS^+^ cells number in IPEC-J2 cells were analyzed by flow cytometry using DCFH-DA. (**B**) Quantification of ROS^+^ cells number in IPEC-J2 cells (*n* = 4). (**C**) The concentration of ROS in IPEC-J2 cells was analyzed by flow cytometry using DCFH-DA. (**D**) Quantification of ROS concentration in IPEC-J2 cells (*n* = 4). The concentration of ROS in IPEC-J2 cells was calculated by the DCF fluorescence intensity. *** *p* < 0.001.

The intracellular ROS level was measured by flow cytometry using DCFH-DA. As shown in Figure 5, TGEV infection increased the number of ROS^+^ cells and the concentration of ROS in IPEC-J2 cells compared with the control group (*p* < 0.001). ATRA treatment significantly inhibited the increasing of ROS^+^ cells number and ROS concentration induced by TGEV, but the number of ROS^+^ cells in TGEV+ATRA group was still higher than that in the control group (*p* < 0.001).

### 3.6. ATRA Attenuated TGEV-Induced Oxidative Stress by Improving Antioxidant Capacity

**Figure 6:**
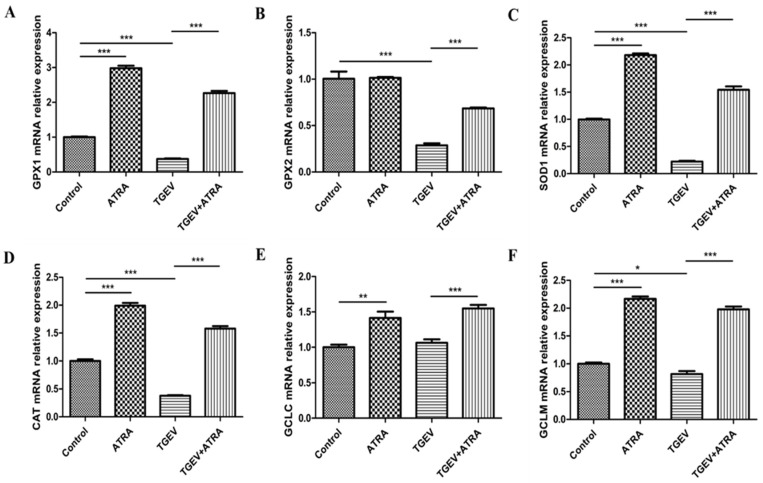
Effects of ATRA on the expressions of antioxidant-related genes in TGEV-infected IPEC-J2 cells. The cells were infected with or without TGEV (1 MOI) for 1 h, followed by incubation with or without 80 μM ATRA for 36 h. (**A**–**F**) The mRNA abundance of GPX1, GPX2, SOD1, CAT, GCLC and GCLM in IPEC-J2 cells were analyzed by real-time PCR (*n* = 4). * *p* < 0.05, ** *p* < 0.01, and *** *p* < 0.001.

**Figure 7:**
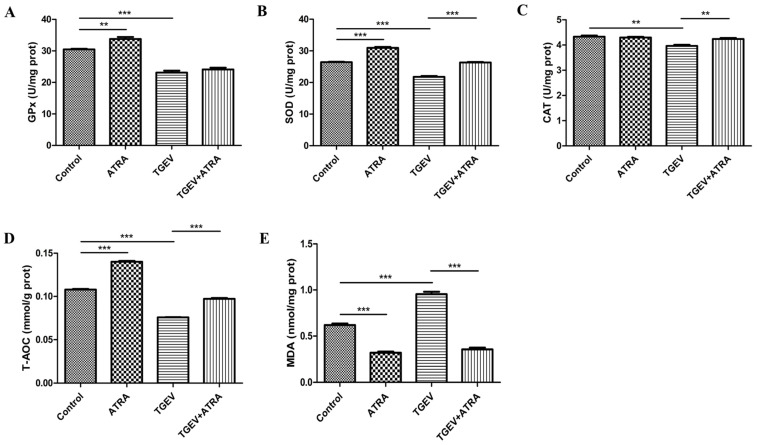
Effects of ATRA on the antioxidant enzyme activities and MDA content in TGEV-infected IPEC-J2 cells. The cells were infected with or without TGEV (1 MOI) for 1 h, followed by incubation with or without 80 μM ATRA for 36 h. The activities of GPx (**A**), SOD (**B**), CAT (**C**) and T-AOC (**D**) and the content of MDA (**E**) were measured by the commercial kits (*n* = 4). ** *p* < 0.01 and *** *p* < 0.001.

To investigate whether ATRA attenuated TGEV-induced oxidative stress by improving the antioxidant capacity, the expressions of antioxidant-related genes were analyzed by real-time PCR. As shown in Figure 6, compared with the control group, the mRNA abundance of GPX1, SOD1, CAT, GCLC, and GCLM were significantly increased in the ATRA group (*p* < 0.01). TGEV infection significantly decreased the mRNA expressions of GPX1, GPX2, SOD1, CAT, and GCLM compared with the control group (*p* < 0.05), whereas ATRA treatment significantly reversed the reduction in GPX1, GPX2, SOD1, CAT, GCLC, and GCLM mRNA expressions induced by TGEV (*p* < 0.001). To further confirm the effects of ATRA on the oxidative stress induced by TGEV, we detected the antioxidant enzyme activities and MDA content in IPEC-J2 cells (Figure 7). Compared with the control group, ATRA alone treatment significantly upregulated the activities of GPx, SOD, and T-AOC, and downregulated the content of MDA (*p* < 0.01). TGEV infection markedly reduced GPx, SOD, CAT, and T-AOC activities, and significantly increased MDA content (*p* < 0.01). However, ATRA treatment significantly blocked the reduction in SOD, CAT, and T-AOC activities, and the increase in MDA content was induced by TGEV (*p* < 0.01).

### 3.7. ATRA Prevented TGEV-Induced P_38_MAPK Signaling Pathway Activation in IPEC-J2 Cells

**Figure 8:**
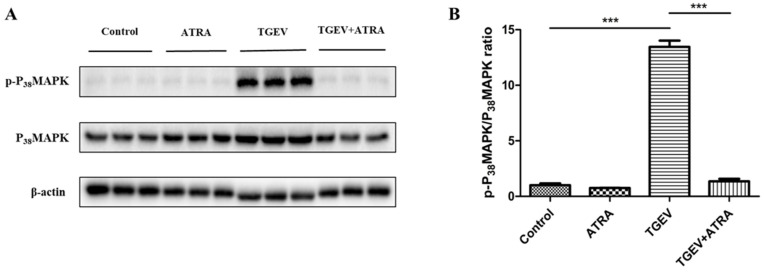
Effects of ATRA on P_38_MAPK pathway in TGEV-infected IPEC-J2 cells. The cells were infected with or without TGEV (1 MOI) for 1 h, followed by incubation with or without 80 μM ATRA for 36 h. (**A**,**B**) The phosphorylation level of P_38_MAPK was analyzed by Western blot (*n* = 3). *** *p* < 0.001.

As shown in Figure 8, the phosphorylation level of P_38_MAPK was significantly upregulated in TGEV-infected IPEC-J2 cells compared with the control group (*p* < 0.001). However, ATRA treatment significantly prevented the upregulation of P_38_MAPK phosphorylation level induced by TGEV (*p* < 0.001).

### 3.8. ATRA Attenuates TGEV-Induced Apoptosis in IPEC-J2 Cells via Inhibiting the ROS-Mediated P_38_MAPK Signaling Pathway

**Figure 9:**
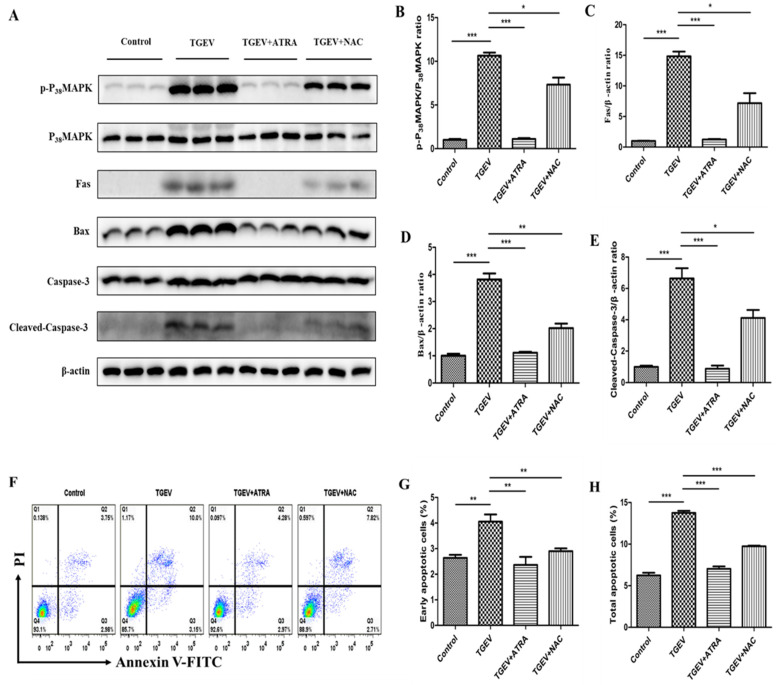
ATRA attenuated TGEV-induced apoptosis in IPEC-J2 cells via inhibiting the ROS-mediated P_38_MAPK signaling pathway. The cells were pre-treatment by ROS inhibitor (NAC, 10 mM) for 2 h followed by infection with TGEV (1 MOI) for 1 h, then incubation with NAC or 80 μM ATRA for 36 h. (**A**–**E**) The phosphorylation level of P_38_MAPK and the protein levels of Fas, Bax and Cleaved-caspase-3 were analyzed by western blot (*n* = 3). (**F**) Annexin V-FITC/PI staining cells were analyzed by flow cytometry. (**G**) Quantification of the percentages of early apoptotic cells (Q3 quadrant, *n* = 4). (**H**) Quantification of the percentages of total apoptotic cells (Q3+Q2 quadrants, *n* = 4). * *p* < 0.05, ** *p* < 0.01, and *** *p* < 0.001.

To further investigate whether ATRA attenuated TGEV-induced apoptosis via inhibiting the ROS-mediated P_38_MAPK signaling pathway, the effect of ATRA was examined by using ROS inhibitors (N-Acetyl-L-cysteine, NAC). As shown in Figure 9A–E, NAC treatment significantly inhibited the enhancing of P_38_MAPK phosphorylation level and the upregulation of Fas, Bax, and Cleaved-caspase-3 protein levels induced by TGEV (*p* < 0.05). Furthermore, we also found that NAC treatment significantly suppressed the elevation of the number of early and total apoptotic IPEC-J2 cells induced by TGEV (*p* < 0.01) (Figure 9F–H). However, the levels of these measured parameters were still higher in TGEV + NAC group than in the control and TGEV + ATRA group, suggesting that ATRA may also attenuate TGEV-induced apoptosis through other pathways.

## 4. Discussion

Transmissible gastroenteritis virus (TGEV), a member of the coronavirus family, can infect pigs of all ages and cause diarrhea, dehydration, and high mortality in piglets, and causes significant losses to the pig industry [1]. Intestinal epithelial cells are the primary target sites for TGEV infection. In vivo, TGEV replicates in the small intestinal epithelial cells of piglets, causing villus atrophy and crypt hyperplasia, and impairing intestinal barrier integrity [32]. Furthermore, TGEV infection also induces IPEC-J2 cells damage by down-regulating the levels of proteins involved in tight and adhesion junctions (ZO-1, Occludin and E-cadherin) in vitro [33]. All-trans retinoic acid (ATRA) is an active metabolite of VA and plays important roles in antioxidant, antiviral, regulating immune response, and improving intestinal barrier function [24,34,35]. However, it is unclear whether ATRA can alleviate IPEC-J2 cell damage caused by TGEV. It is known that the release of LDH is an important indicator to assess the severity of cell damage. In the present study, we found that TGEV infection significantly increased the LDH activity in IPEC-J2 cell culture medium, whereas ATRA treatment significantly reduced the activity of LDH in TGEV-infected IPEC-J2 cell culture medium in a dose-dependent manner. To further determine the protective effects of ATRA on the TGEV-induced IPEC-J2 cells damage, we detected tight junction-associated proteins (ZO-1, Occludin, and Claudin-2) mRNA expressions. Tight junction proteins and mucin proteins are considered important regulators for maintaining the structural and functional integrity of the intestinal barrier [36]. He et al. reported that ATRA significantly reversed LPS-induced IPEC-J2 cells damage via enhancing the expressions of ZO-1, Occludin, and Claudin-1 [37]. Our results found that ATRA treatment significantly suppressed the downregulation of ZO-1, Occludin, and Mucin-1 mRNA expressions induced by TGEV. These results indicated that ATRA can attenuate TGEV-induced IPEC-J2 cell damage. Claudin-2 has been shown to be associated with epithelial barrier leakage and diarrhea. However, our results found that TGEV infection significantly downregulated the mRNA expression of Claudin-2, whereas ATRA treatment significantly reversed this downregulation of Claudin-2 mRNA expression induced by TGEV. The specific reason is still unclear and needs further study.

Apoptosis is the main component that contributes to the pathogenesis of viral infectious diseases in humans and animals [38]. Previous studies reported that TGEV infection could trigger cell apoptosis and further lead to CPE and cell death of host cells [10,11,12]. The evidence of TGEV-induced apoptosis indicates that apoptosis is involved in the pathogenesis of TGEV. Our results also found that TGEV infection induced CPE and apoptosis in IPEC-J2 cells. However, ATRA treatment could evidently reverse the CPE and apoptosis induced by TGEV. Similar results also reported that ATRA treatment significantly suppressed the CPE and apoptosis in PC12 cells following oxygen and glucose deprivation injury [39]. These results indicated that ATRA may attenuate TGEV-induced IPEC-J2 cells damage via suppressing apoptosis. Caspases are a family of cysteine-dependent aspartate-directed proteases, which play a key role in the execution of apoptosis [40]. Caspase-3 is one of the most common apoptosis executioners. Previous studies have shown that the activation of caspases-3 plays a key role in apoptosis induced by TGEV [41]. Our results found that TGEV infection significantly increased the Cleaved-caspase-3 protein level and Caspase-3 mRNA expression. However, ATRA treatment significantly inhibited the elevation of Cleaved-caspase-3 protein level and Caspase-3 mRNA expression induced by TGEV. These results indicated that ATRA may attenuate TGEV-induced IPEC-J2 cells apoptosis via suppressing caspase-3 mediated apoptosis pathway.

Caspase-3 is activated mainly through two pathways: the extrinsic (death receptor pathway) and intrinsic pathway (mitochondrial pathway) [42]. The death receptor pathway is initiated by the ligation of death receptors (Fas) and subsequent caspase-8 activation [43]. The mitochondrial pathway is initiated by the increase in mitochondrial membrane permeability, leading to the activation of caspase-9 [44]. Bax and Bcl-2 are members of the Bcl-2 family proteins and are the major regulators that regulate the integrity of mitochondrial membranes [45]. Ding et al. reported that TGEV infection could induce PK-15 cell apoptosis through activating death receptor and mitochondrial-mediated apoptotic pathways [31]. In this study, we found that TGEV infection significantly upregulated Fas, Bax, Caspase-8, and Caspase-9 mRNA expressions, increased Fas, Cleaved-caspase-8, and Cleaved-caspase-9 protein levels and the ratio of Bax/Bcl-2 in IPEC-J2 cells, while ATRA treatment significantly prevented the upregulation of Fas, Bax, and Caspase-8 mRNA expressions and the increasing of Fas, Cleaved-caspase-8, and Cleaved-caspase-9 protein levels and Bax/Bcl-2 ratio induced by TGEV. These results indicated that ATRA may attenuate TGEV-induced IPEC-J2 cells apoptosis by inhibiting the activation of death receptor and mitochondrial-mediated apoptotic pathways.

To further study the mechanism by which ATRA inhibit TGEV-induced IPEC-J2 cell apoptosis, we detected the level of ROS in IPEC-J2 cells. Studies have shown that both death receptor-mediated apoptosis and mitochondrial-mediated apoptosis are closely related to oxidative stress induced by ROS [46,47]. A number of studies have reported that ROS is involved in virus-induced apoptosis [15,16]. Hong et al. reported that TGEV infection induced PK-15 cells apoptosis via mediating ROS accumulation [17]. Our results also found that TGEV infection significantly increased the accumulation of ROS in IPEC-J2 cells. However, ATRA treatment significantly inhibited the increasing of ROS^+^ cells number and ROS concentration induced by TGEV. These results indicated that ATRA may attenuate TGEV-induced IPEC-J2 cells apoptosis via suppressing ROS production. This may be related to the antioxidant properties of ATRA. Rao et al. reported that ATRA alleviated hepatic ischemia/reperfusion injury by enhancing the activity of SOD and inhibiting MDA formation in rats [24]. Khafaga et al. reported that ATRA ameliorated doxorubicin induced cardiac oxidative damage of the rats by enhancing the activities of antioxidant enzymes, such as GPx, SOD, and CAT, and decreasing the MDA concentration [25]. Similar to these reports, our results found that ATRA treatment significantly increased the activities of SOD, CAT, and T-AOC, and decreased the MDA content in TGEV-infected IPEC-J2 cells. These results indicated that ATRA attenuated TGEV-induced oxidative stress by improving antioxidant capacity. To further confirm whether ATRA attenuated TGEV-induced oxidative stress by improving antioxidant capacity, we detected the expressions of antioxidant-related genes in IPEC-J2 cells. Our results found that ATRA treatment significantly reversed the decrease in mRNA expressions of antioxidant-related genes (GPX1, GPX2, SOD1, CAT, GCLC, and GCLM) induced by TGEV. The above results indicated that ATRA can attenuate TGEV-induced ROS production by improving the antioxidant capacity, thereby inhibiting the apoptosis of IPEC-J2 cells.

Furthermore, ROS is also related to some apoptosis signaling pathways, such as MAPK and p53 pathways [18]. Ding et al. reported that TGEV infection induced PK-15 cells apoptosis by activating ROS-mediated p53 and P_38_MAPK signaling pathways [20]. To investigate whether the P_38_MAPK signaling pathway is involved in the process of ATRA attenuating TGEV-induced IPEC-J2 cells apoptosis, we assessed the phosphorylation level of P_38_MAPK by Western blot. Our results found that the phosphorylation level of P_38_MAPK was significantly upregulated in TGEV-infected IPEC-J2 cells. However, ATRA treatment prevented the upregulation of the P_38_MAPK phosphorylation level induced by TGEV. These results indicated that the inhibition of P_38_MAPK signaling pathway plays an important role in ATRA attenuating TGEV-induced IPEC-J2 cells apoptosis. To further confirm whether ATRA attenuated TGEV-induced apoptosis via inhibiting the ROS-mediated P_38_MAPK signaling pathway, we used the ROS-specific inhibitors (NAC) to inhibit ROS production in TGEV-infected IPEC-J2 cells. Our results found that NAC treatment significantly inhibited TGEV-induced P_38_MAPK activation. Next, we further determine the effects of NAC on TGEV-induced IPEC-J2 cells apoptosis. Our results found that NAC treatment significantly decreased the protein levels of pro-apoptotic protein Fas, Bax, and Cleaved-caspase-3 and the percentage of apoptotic cells in TGEV-infected IPEC-J2 cells. The above results suggested that ATRA attenuated TGEV-induced IPEC-J2 cells apoptosis via inhibiting the ROS-mediated P_38_MAPK signaling pathway.

## 5. Conclusions

**Figure 10:**
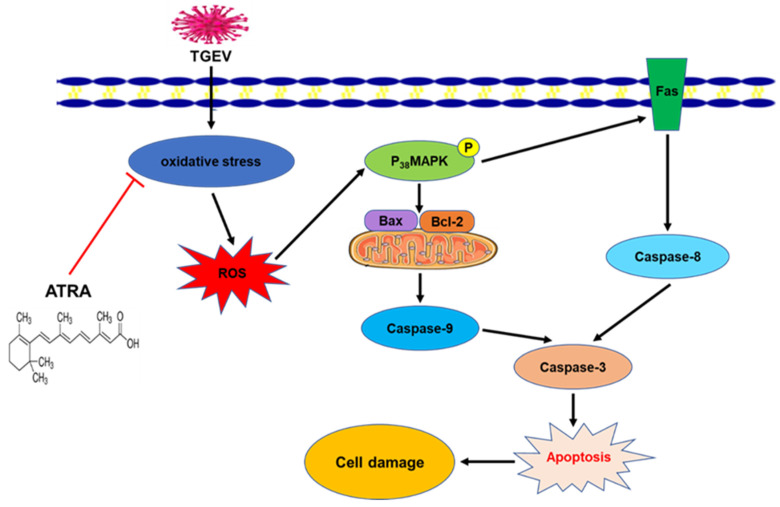
Proposed model of the protective effect of ATRA in TGEV-induced oxidative stress and apoptosis. (1) ATRA attenuated TGEV-induced oxidative stress by modulating the activities of antioxidant enzymes. (2) ATRA attenuated TGEV-induced apoptosis via inhibiting the activation of death receptor and mitochondrial-mediated apoptotic pathways, which is associated with the inhibition of ROS-mediated P_38_MAPK signaling pathway.

In conclusion, our results indicated that ATRA attenuated TGEV-induced apoptosis in IPEC-J2 cells via improving the antioxidant capacity, thereby inhibiting the cell damage. The mechanism is associated with the inhibition of ROS-mediated P_38_MAPK signaling pathway (Figure 10). These results help us to better understand the pathogenesis of TGEV and provide a new strategy for the treatment of TGEV infection.

## Data Availability

Data presented in this study are presented in the article and Supplementary Material.

## Supplementary Materials

The following are available online at https://www.mdpi.com/article/10.3390/antiox11020345/s1, Table S1: Primer sequences used for real-time PCR.
