# Supplementary material for: All-Trans Retinoic Acid Attenuates Transmissible Gastroenteritis Virus-Induced Apoptosis in IPEC-J2 Cells via Inhibiting ROS-Mediated P38MAPK Signaling Pathway
Source: Antioxidants (Basel). 2022 Feb 10;11(2):345. doi: 10.3390/antiox11020345 (PMC8868330; doi:10.3390/antiox11020345)
Supplement: Supplementary file 1 [file antioxidants-11-00345-s001.zip › antioxidants-1546747-supplementary.pdf]

Supplemental Table S1. Primer sequences used for real-time PCR.

Gene	Primer Sequence (5′ –3′)	Product Length(bp)	GeneBank Accession No.
ZO-1	F: CGTGTCAACGCCACTATCA<br>R: TTGTCTTCCAAAGCCCCT	105	XM_021098896.1
Occludin	F: AACTTCCACTGATGTCCCCCGT<br>R: CCTAGACTTTCCTGCTCTGCCC	116	NM_001163647.2
Claudin-2	F: GCATCATTTCCTCCCTGTT<br>R: TCTTGGCTTTGGGTGGTT	156	NM_001161638.1
Mucin-1	F: GTGCCGCTGCCCACAACCTG<br>R: AGCCGGGTACCCCAGACCCA	141	XM_021089730.1
Mucin-2	F: GGTCATGCTGGAGCTGGACAGT<br>R: TGCCTCCTCGGGGTCGTCAC	181	XM_021082584.1
Fas	F: TGATGCCCAAGTGACTGACC<br>R: GCAGAATTGACCCTCACGAT	103	NM_213839
Bax	F: GACGCTGGACTTCCTTCGAG<br>R: GTGGCCCGAGAGAGGTTTATT	334	XM_013998624.2
Bcl-2	F: GCTACTTACTGCCAAAGGGA<br>R: TTCAGGCGGAGCTGTAAGAG	161	XM_021099593.1
Caspase-3	F: GGAATGGCATGTCGATCTGGT<br>R: ACTGTCCGTCTCAATCCCAC	351	NM_214131.1
Caspase-8	F: TCTGCGGACTGGATGTGATT<br>R: TCTGAGGTTGCTGGTCACAC	165	XM_021074714.1
Caspase-9	F: AATGCCGATTTGGCTTACGT<br>R: CATTTGCTTGGCAGTCAGGTT	195	XM_013998997.2
GPX1	F: GTGAATGGCGCAAATGCTCA<br>R: ATTGCGACACACTGGAGACC	126	NM_214201.1
GPX2	F: AGAATGTGGCCTCGCTCTGA<br>R: GGCATTGCAGCTCGTTGAG	112	DQ898282
SOD1	F: AGACCTGGGCAATGTGACTG<br>R: GTGCGGCCAATGATGGAATG	102	NM_001190422.1
CAT	F: CGAAGGCGAAGGTGTTTG<br>R: AGTGTGCGATCCATATCC	132	NM_214301.2
GCLC	F: TCCTCCTCCAAACTCCGACA<br>R: GCACCACAAACACCACGTAC	101	XM_021098556.1
GCLM	F: GTGATGCCGCCCGATTTAAC<br>R: TCATGTGCCTCGATGTCAGG	140	XM_001926378.4
β-actin	F: GGATGACGATATTGCTGCGC<br>R: GATGCCTCTCTTGCTCTGGG	190	XM_003124280.5

ZO-1, zonula occludens 1; *Bax*, B-cell lymphoma-2-associated X protein; *Bcl-2*, B-cell lymphoma-2; *GPX*, glutathione peroxidase; *SOD1*, superoxide dismutase 1; *CAT*, catalase; *GCLC*, catalytic subunit of glutamate-cysteine ligase; *GCLM*, modifier subunit of glutamate-cysteine ligase
